# Oral Health-Related Quality of Life Improvement After Treatment With Fixed and Removable Dental Prostheses

**DOI:** 10.7759/cureus.71013

**Published:** 2024-10-07

**Authors:** Arwa U AlSaggaf, Alaa Alqutub, Zyad Almasri, Faisal Khalifah, Faris Khuzaee, Abdulmohsen Aljuaid, Omair Bukhari, Amin A Marghalani

**Affiliations:** 1 Oral and Maxillofacial Surgery Department, College of Dental Medicine, Umm Al-Qura University, Makkah, SAU; 2 College of Dental Medicine, Umm Al-Qura University, Makkah, SAU; 3 Preventive Dentistry Department, College of Dental Medicine, Umm Al-Qura University, Makkah, SAU

**Keywords:** complete dentures, fixed partial dentures, oral health, quality of life, removable partial dentures, single crowns

## Abstract

Background: Missing teeth negatively affect oral functions and masticatory efficiency, which in turn can reduce a person's quality of life (QoL). This study is aimed at evaluating the effect of dental prostheses on oral health-related quality of life (OHRQoL) in adults.

Materials and methods: This is an observational study to measure patients' OHRQoL using a cohort study design before and after treatment with different dental prostheses (fixed and removable). Ninety-seven patients who received dental prostheses responded to a questionnaire before dental prosthetic treatment and then after the completion of the treatment. The conduction was face-to-face or via telephone interviews. The questionnaire is divided into two parts to verify the demographic and the patient's related factors and to measure OHRQoL using the Oral Health Impact Profile-5 (OHIP-5).

Results: Descriptive statistics, inferential statistics, and multivariable analysis were conducted using Stata Statistical Software: Release 14.2 (2016; StataCorp LLC, College Station, Texas, United States) and were at a 0.05 significance level. Data were collected from the 97 patients, of which 70% were male with a mean age of 39.5 and 30% were female with a mean age of 43. The educational level showed that only 15% of the patients received a bachelor's degree and that the remaining 85% stopped at a high school general educational level. The received prosthetic treatment consisted of 23% single crowns, 29% fixed partial dentures (FPD), 20% removable partial dentures (RPD), and 28% complete dentures. Self-reported overall health indicated that 64% of the patients had very good health and 36% had good health or a lower quality of health. There was a significant difference in OHIP scores before prosthetic treatment (M=5.4, SD=0.57) and after prosthetic treatment (M=3.2, SD=0.49) with no significant difference in the reduction in OHIP scores between fixed prostheses (M=−2.8, SD=0.64) and removable prostheses (M=−1.5, SD=0.74). The amount of improvement in OHIP answers after wearing a prosthesis was significant (p<0.0001). Linear regression indicated that having a history of discomfort from general oral health improved (decreased OHIP score) by 2.2 after prosthetic dental treatment compared to participants without a history of general oral health discomfort when all other variables are held constant.

Conclusion: Within the limitations of this study, it can be concluded that restoration of missing teeth with a dental prosthesis has an immense impact on individuals' QoL, regardless of the type of dental prosthetic restoration.

## Introduction

Over the past 30 years, health-related quality of life (QoL) measures have been established to aid in patient-based health assessment for integration into the decision-making process [1]. The World Health Organization (WHO) defines health as a full condition of physical, mental, and social well-being, rather than simply the absence of sickness [2]. QoL is defined as individuals' perceptions of their place in life in relation to the culture and value systems in which they live, as well as their goals, expectations, standards, and worries [3].

Oral health has been part of the developed assessment measure as oral health-related quality of life (OHRQoL), which showed widespread use in research and practice fields to evaluate the needs of oral treatment, oral health, and the dental treatment outcomes that impact every aspect of daily activities [4,5]. OHRQoL is a fundamental part of general health-related QoL because of its impact on patients' expectations, experiences, and sociocultural backgrounds [6,7].

Several OHRQoL measures are utilized in clinical and public health research. However, the Oral Health Impact Profile (OHIP) is the most suitable and widely used tool for assessing OHRQoL [1,8]. The five-item OHIP comprises five questions that represent four specified aspects: oral function, orofacial pain, orofacial appearance, and psychosocial effects. Although the OHIP only contains 10% of the original OHIP, it can collect over 90% of the essential data. Therefore, it is the most accepted and attractive tool for assessing OHRQoL [9].

Oral health is an important factor for general health [10]. Evidence implies that oral disease is equally as important as other diseases for individuals' QoL and their psychosocial and emotional status, including isolation, depression, and unemployment [11]. Patients with low education levels, anxiety, and poor general health are positively associated with poor oral health. It is also reported that there is an increase in the prevalence of oral disease among elderly patients [12,13].

Oral functions and masticatory efficiency are severely affected by tooth loss [14]. Tooth loss can be caused by many reasons; however, dental caries and periodontal diseases are the most common cause [15,16]. Difficulty eating due to partial or complete edentulism is correlated to poor OHRQoL in prior studies [17]. Some patients from various educational and socioeconomic backgrounds do not seek treatment promptly following tooth loss, even if replacement is strongly indicated, due to a lack of information about the significance of prosthodontic replacement, financial concerns, lack of time, or lack of motivation [18].

However, most populations exhibit awareness of prosthodontic treatment. Patients' factors such as age, gender, socioeconomic status (SES), educational level, preferences, and esthetic concerns have predominantly influenced the preferred prosthodontic treatments [14]. It is reported that the majority of patients are concerned with restoring missing anterior teeth more than posterior teeth and that aesthetic demands are higher than functional needs [19]. In addition, patients have been reported to prefer fixed partial dentures (FPD) over removable partial dentures (RPD) to replace missing teeth [20]. According to Akeel, 82% of Saudi Arabian male patients have a desire to restore missing teeth [21]. This study aimed to measure the patient's QoL before and after restoring their missing teeth with FPD or RPD by assessing OHRQoL measurements.

## Materials and methods

This study is aimed at observing and measuring patients' QoL through a cohort study design (prospective) before and after dental prosthesis treatment. Ethical approval was obtained from the Biomedical Research Ethics Committee of Umm Al-Qura University in Makkah, Saudi Arabia (approval number: HAPO-02-K-012-2022-02-969).

This cohort study of patients receiving dental prostheses was designed to measure OHRQoL in patients receiving different dental prostheses (fixed and removable) by measuring the before and after treatment scores. The participants are patients (regardless of gender and age) visiting the Dental Teaching Hospital who require a dental prosthesis, crown, FPD, RPD, and/or full denture. Eligible participants received participants' information sheets (PIS) and consented.

A sample size of 60 achieves 90% power to detect an R-squared value of 0.15, which is attributed to one independent variable using an F-test with a significance level (alpha) of 0.05. The tested variables are adjusted for an additional six independent variables with an R-squared value of 0.29. The sample size reached in this study is 97 patients. The patients who were willing to participate in the study were either interviewed face-to-face if data collectors saw them in the clinic or via telephone interviews for those who could not be interviewed during their visit to the hospital. The interview was structured by using a questionnaire before dental prosthetic treatment and then after completion of the treatment. The questions were divided into two parts.

The first part is divided into two sections. The first section comprises questions about demographic variables (i.e., age, gender, marital status, and education), income-related factors (i.e., number of family members who support the family and the average income), general health status, and type of dental prosthesis. Types of prostheses include complete denture, RPD (upper/lower), bridge, or crown. In the second section, patients were asked about their chief complaints and satisfaction. Following the previous questions, the patients were asked if they felt any discomfort about their missing teeth or felt any pain or sensitivity and wanted to restore them.

The second part is the participants' OHRQoL, which was measured using a validated Arabic version of OHIP-5 (OHIP5-Ar) that has been previously utilized [9]. OHIP5-Ar includes five closed-ended questions, and the range of answers in the tool is listed as follows: (1) very often, (2) fairly often, (3) occasionally, (4) hardly ever, or (5) never [9].

The five closed-ended questions were the following: (1) Have you had any difficulty chewing food because of problems with teeth, mouth, denture, or jaw? (2) Have you had any painful aching in your mouth? (3) Have you felt uncomfortable about the appearance of your teeth, mouth, denture, or jaw? (4) Have you felt that there has been less flavor in your food because of problems with your teeth, mouth, denture, or jaw? (5) Have you had difficulty performing your everyday activities because of problems with your teeth, mouth, denture, or jaw?

Descriptive statistics, including the mean, standard deviation, frequency, and percentages, were reported. For categorical variables, the frequencies and percentages were reported to provide a relative perspective on the variable's composition. Inferential statistics were calculated using a paired sample t-test to compare OHIP scores before and after prosthetic treatments. Furthermore, a two-sample t-test was performed to compare the reduction in OHIP scores between different categorical variables.

For multivariable analysis, stepwise linear regression analysis was conducted to determine the significant predictors of the OHIP change. The analysis started with a complete model that comprises all potential independent variables while forcing the model to include socio-demographic variables such as age, gender, and marital status. Throughout the stepwise regression, a probability of entry of 0.1 and a probability of removal of 0.2 were applied. All statistical analyses were performed using Stata Statistical Software: Release 14.2 (2016; StataCorp LLC, College Station, Texas, United States) and were at the 0.05 significance level.

## Results

Data were collected from 97 patients, of which 68 (70%) were male with a mean age of 39.5 and 29 (30%) were female with a mean age of 43. The marital status for the sample patients was 60 (62%) married and 37 (38%) single, while the educational level showed that only 15 (15%) of the patients received a bachelor's degree and that the remaining 82 (85%) stopped at a high school general educational level. Data on last year's annual household income showed that 81 (84%) of the patients had less than 40K Saudi Arabian riyal (SAR) income, while 16 (16%) of the patients had more than 40K SAR income.

Single crowns represented 23% (22) of all prosthesis treatments, FPD represented 28 (29%) of all prosthesis treatments, RPD represented 20 (20%) of all prosthesis treatments, and complete dentures represented 27 (28%) of all prosthesis treatments. Self-reported overall health indicated that 62 (64%) of the patients had very good health and that 35 (36%) of the patients had good health or lower quality of health (Table 1).

**Table 1:** Demographical data SD: standard deviation; SAR: Saudi Arabian riyal; FPD: fixed partial denture; RPD: removable partial denture

Demographic factor		No. (%)	Mean age	SD
Gender	Male	68 (70%)	43	17.1
	Female	29 (30%)	39.5	15.6
Marital status	Married	60 (62%)	48.5	14.1
	Single	37 (38%)	27.1	8.4
Education	High school general education	82 (85%)	41.6	16.3
	Bachelor's degree	15 (15%)	34.5	13.5
Annual household income last year	Less than 40K SAR	81(84%)	40.9	16.1
	40K SAR or more	16 (16%)	38.7	15.8
Prosthesis type	Crown	22 (23%)	31	16.5
	FPD	28 (29%)	31.4	10
	RPD	20 (20%)	49.2	15
	Full denture	27 (28%)	51.9	10.5
Removable vs. fixed prosthesis	Fixed	50 (52%)	31.3	13.1
	Removable	47 (48%)	50.8	12.4
Overall self-reported health	Very good	62 (64%)	38	15.2
	Good or lower	35 (36%)	45.4	16.6
Total (100%)			40.6	16

**Figure 1:**
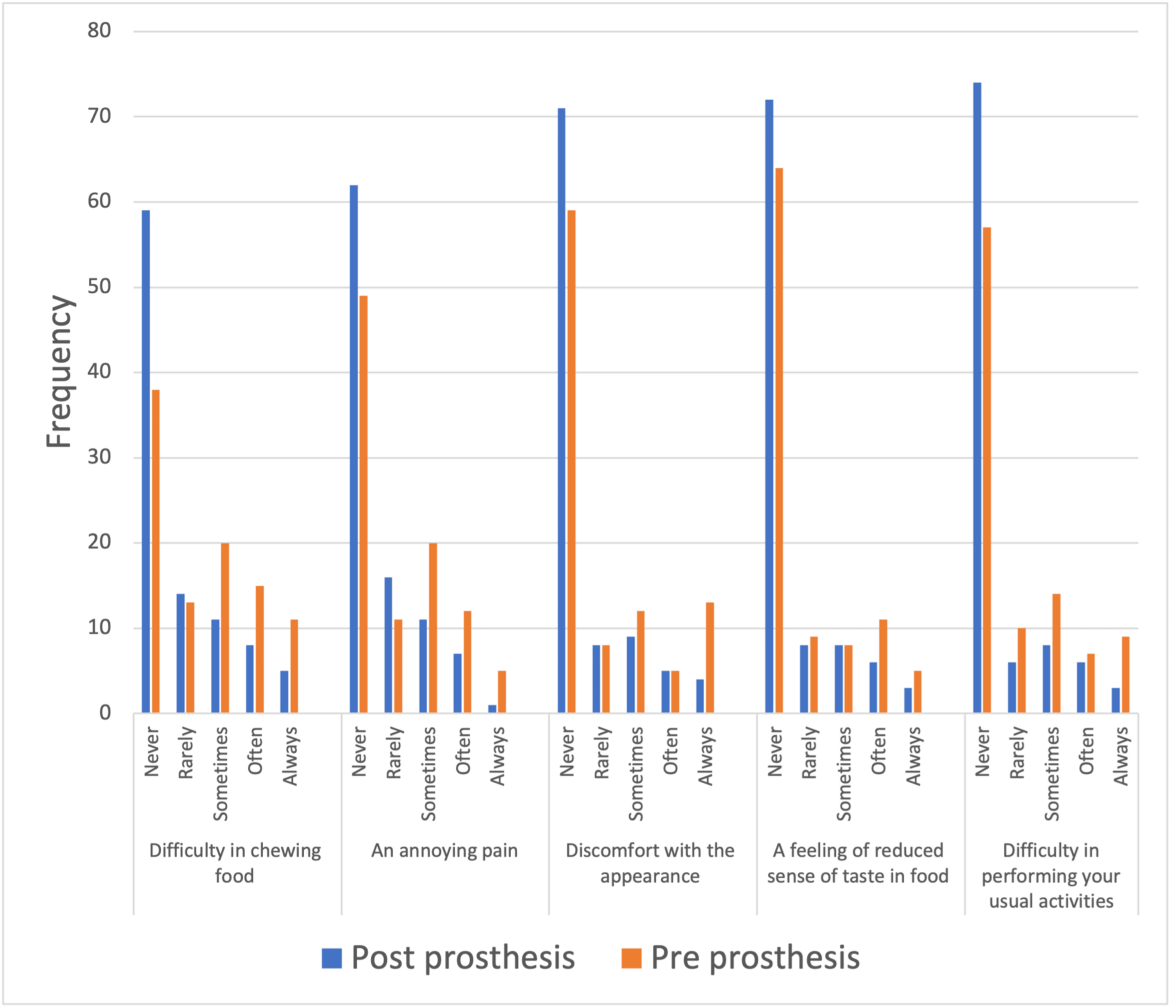
OHIP before and after wearing a dental prosthesis OHIP: Oral Health Impact Profile

There was a significant difference in OHIP scores between before prosthetic treatment (M=5.4, SD=0.57) and after prosthetic treatment (M=3.2, SD=0.49) (p<0.0001) (Figure 1). There was no significant difference in the reduction in OHIP scores between fixed prostheses (M=−2.8, SD=0.64) and removable prostheses (M=−1.5, SD=0.74) (p=0.1923). The amount of improvement in OHIP answers after wearing a prosthesis was significant (p<0.0001 using a paired t-test).

Linear regression indicated that having a history of general oral health discomfort improved (decreased) the OHIP score by 2.2 after prosthetic dental treatment compared to participants without a history of general oral health discomfort when all other variables were held constant (Table 2).

**Table 2:** Results of linear regression to predict OHIP change using the following variables while adjusting for age and gender SAR: Saudi Arabian riyal; OHIP: Oral Health Impact Profile

	Coef.	St. err.	P-value	95% conf. interval
Age	0.011	0.04	0.789	−0.069-0.09
Gender (male)	−1.57	1.078	0.15	−3.708-0.576
Marital status (single)	−0.57	1.332	0.67	−3.215-2.077
Education (undergraduate)	−0.82	1.421	0.565	−3.645-2.002
Income (40K SAR or more)	−0.66	1.404	0.638	−3.453-2.127
History of discomfort from general oral health (yes)*	−2.21	1.012	0.032	−4.215-0.196
R-squared	0.101	Number of observations		97
F-test	1.681	Prob >F		0.135

## Discussion

In analyzing the effect of dental prostheses on OHRQoL among adults in Saudi Arabia, the results based on primary research with 97 patients who received dental prostheses and responded to the questionnaire before and after the completion of their treatment provide interesting insights.

The first noteworthy finding is that there is a distinct improvement in OHIP before and after undergoing treatment with a dental prosthesis. For instance, when asked about difficulty chewing food, 38 (or 39%) of the respondents pre-prosthesis stated they never had difficulty chewing food. Post-prosthesis, 59 (or 61%) of respondents confirmed that they never had difficulty chewing food. Eleven (or 11.3%) of respondents pre-prosthesis stated that they always had difficulty chewing food, which reduced to 4% of the patients who had prosthesis treatment. Almohsen and Mahmoud investigated patient satisfaction with RPD in the Qassimi region of Saudi Arabia and reported that after receiving RPDs, there was a reduction in difficulty chewing by 26.7% as observed among participants [22]. This finding confirms the effectiveness of a dental prosthesis in adding to the overall convenience when chewing food.

With regard to annoying pain, 49 (or 50%) of the respondents pre-prosthesis stated that they never had annoying pain, increasing to 63% of the respondents post-prosthesis. The tendency to always have annoying pain also reduced from 5% of pre-prosthesis patients to only 1% of patients post-prosthesis, which reinforces the effectiveness of prosthesis treatment in minimizing annoying dental pain. Alzoubi et al. investigated the management of pain associated with orthodontic treatment in Saudi Arabia and also established that a prosthesis was associated with a reduction in pain reported by patients [23]. The authors referred to two key reasons for this reduction in annoying pain. The first reason is the protection of damaged teeth through the use of techniques such as dental crowns to serve as protective covers, which help shield the teeth from further damage. The second reason is the resolution of infection. Prosthesis treatment helps restore teeth and eliminate infection, thereby relieving pain.

Discomfort with one's appearance is also substantially reduced after undergoing prosthesis treatment. For instance, 12 (12%) of the participants reported discomfort with their appearance before prosthesis treatment, which reduced to only 3% of the participants post-prosthesis, whereas 70 (73%) of the participants post-prosthesis reported they never experienced discomfort with their appearance, an increase from 59 (61%) of participants. Alshammari et al. evaluated the OHRQoL among the elderly with edentulous jaws in the Hafar Al-Batin region of Saudi Arabia and reported that a dental prosthesis had a positive association with OHRQoL. Specifically, a dental prosthesis reduced psychological and physical discomfort experienced by the participants, which reinforces the findings of my study [24].

The difference between pre- and post-prosthesis responses from participants regarding the feeling of a reduced sense of taste in food is not significant (as illustrated in Figure 1), a finding supported by Ganesan et al. [25]. Regarding difficulty performing usual activities, nine (or 9.3%) of the respondents pre-prosthesis stated that they had difficulty performing their usual activities. It reduced to only 2% of the respondents post-prosthesis, which illustrates the reduction in difficulty performing usual activities once the participants had received prosthesis treatment, thereby indicating the effectiveness of prosthetic dental treatment in helping them perform their usual activities. The relevance of a dental prosthesis in lowering the difficulty performing usual activities was also reported by El-Kholey et al. and Felemban et al., both of whom reported a statistically significant reduction in difficulty performing usual activities, as reported by the participants [26,27]. The usefulness of prosthetic dental treatment in reducing discomfort and pain, lowering the difficulty associated with performing usual activities, and reducing the difficulty chewing food is also reported by Akeel, Tsakos et al., and Khan and Ghani [14,17,21].

The second interesting insight gained via the primary research pertains to the regression analysis conducted to predict OHIP change based on the different variables. The history of discomfort from general oral health was found to have a statistically significant effect in improving (reducing) the OHIP score by 2.2 units after receiving the prosthetic dental treatment compared to the participants who did not have a history of discomfort from general oral health (Table 2). This finding highlights that for those with prior discomfort associated with general oral health, prosthetic dental treatment is an effective solution in improving their OHRQoL, a finding also noted by Slade and Spencer and Alhajj et al. [5,9].

It is important to pay attention to the demographic background of participants who received a dental prosthesis and completed the questionnaire. Representation of various types of patients with different characteristics improves the reliability and validity of the findings. For instance, the prosthesis type is reasonably well represented, as 28% of the patients required a full crown, 23% of the patients required only a crown, 29% of the patients required FPD, and the remaining 20% of the patients required RPD. These results demonstrate that the pre-prosthesis and post-prosthesis analysis undertaken in this study sufficiently represented the various types of prostheses, thereby improving the reliability and generalizability of the results.

Similarly, regarding fixed prostheses versus removable prostheses, 52% of the patients required fixed prosthesis treatment and 48% required removable prosthesis treatment. This finding further reinforces the representation of both fixed prostheses and removable prostheses to encapsulate the viewpoint of patients requiring different types of prosthesis treatment to improve the generalizability of the findings. The representation of patients based on marital status, education level, gender, annual household income, and their corresponding age also improved the quality of insights generated from this study.

A limitation of this study is that the entire sample size was derived from a single dental educational center, which is not representative of the general population, and therefore the results cannot be generalized. Another limitation is the relatively small sample size which could be improved by including more than a single dental center. The authors recommend further studies to investigate implant-supported prostheses in their evaluation of the impact of prosthesis treatment on QoL.

## Conclusions

Within the limitations of this study, the data suggest that restoration of missing teeth with dental prostheses greatly impacts individuals' QoL, regardless of the type of dental prosthetic restoration. Also, improvement in the OHRQoL score was the same for fixed and removable prostheses with individuals suffering from a history of general oral health discomfort.
